# Dysregulation of Systemic and Mucosal Humoral Responses to Microbial and Food Antigens as a Factor Contributing to Microbial Translocation and Chronic Inflammation in HIV-1 Infection

**DOI:** 10.1371/journal.ppat.1006087

**Published:** 2017-01-26

**Authors:** Zdenek Hel, Jun Xu, Warren L. Denning, E. Scott Helton, Richard P. H. Huijbregts, Sonya L. Heath, E. Turner Overton, Benjamin S. Christmann, Charles O. Elson, Paul A. Goepfert, Jiri Mestecky

**Affiliations:** 1 Department of Pathology, University of Alabama at Birmingham, Birmingham, Alabama, United States of America; 2 Department of Microbiology, University of Alabama at Birmingham, Birmingham, Alabama, United States of America; 3 Department of Medicine, University of Alabama at Birmingham, Birmingham, Alabama, United States of America; 4 Department of Natural Science and Mathematics, Lee University, Cleveland, Tennessee, United States of America; 5 Institute of Immunology and Microbiology, 1^st^ School of Medicine, Charles University, Prague, Czech Republic; Emory University, UNITED STATES

## Abstract

HIV-1 infection is associated with an early and profound depletion of mucosal memory CD4^+^ T cells, a population that plays an indispensable role in the regulation of isotype switching and transepithelial transport of antibodies. In this study, we addressed whether the depletion of CD4^+^ T cell in HIV-1-infected individuals results in altered humoral responses specific to antigens encountered at mucosal surfaces. Comprehensive protein microarray of systemic humoral responses to intestinal microbiota demonstrated reduced IgG responses to antigens derived from Proteobacteria and Firmicutes but not Bacteroidetes. Importantly, intestinal secretions of antiretroviral therapy-treated HIV-1-infected individuals exhibited a significant elevation of IgM levels and decreased IgA/IgM and IgG/IgM ratios of antibodies specific to a variety of microbial and food antigens. The presented findings indicate reduced competence of mucosal B cells for class switch recombination from IgM to other isotypes limiting their capacity to react to changing antigenic variety in the gut lumen. Decreased availability of microbiota-specific IgA and IgG may be an important factor contributing to the translocation of microbial antigens across the intestinal mucosal barrier and their systemic dissemination that drives chronic inflammation in HIV-1-infected individuals.

## Author Summary

Despite significant effort, our understanding of how HIV-1 virus undermines the immune system is limited. HIV-1infection is characterized by extensive damage to intestinal mucosal barrier and translocation of bacteria and microbial products into the systemic circulation. Immune activation induced by microbial products results in chronic inflammation that drives HIV-1 pathogenesis and progression to AIDS despite successful control of HIV-1 replication by antiretroviral therapy. Here we provide evidence indicating that antibody-producing cells in the intestinal mucosa of HIV-1-infected individuals have decreased capacity to switch from the production of IgM to IgA and IgG, the types of antibodies that normally restrict the translocation of bacterial antigens across the mucosal barrier. This may facilitate systemic dissemination of microbial products and subsequent inflammation in HIV-1-infected individuals. Furthermore, the accumulation of IgM in gut mucosa may contribute to the exacerbation of inflammatory processes by the formation of inflammatory immune complexes. This newly identified dysregulation of immune system alters our understanding of HIV-1 pathogenesis and may facilitate the design of novel therapies targeting immune dysfunction in HIV-1/AIDS.

## Introduction

Compartmentalization of systemic and mucosal immunity restricts adaptive immunity to intestinal microbiota by a complex system of physical and bioactive barriers. This compartmentalization is altered in conditions associated with increased gut epithelial permeability resulting in systemic immune responses to intestinal microbial antigens [1–6]. Massive depletion of CD4^+^ T cells in lymphoid tissue, most profoundly in gut-associated lymphoid tissue (GALT), in the first weeks of HIV-1 infection sets the overall course of the ensuing disease [7–10]. The extent of this initial hit to the mucosal immune system appears to determine disease progression; in this sense, first battle decides the war [11]. Accumulated evidence demonstrates that HIV-1 and simian immunodeficiency virus (SIV) cause extensive damage to the gastrointestinal mucosal surface, epithelial microenvironment, and antimicrobial functions of the mucosal barrier [8, 12, 13]. Elevated microbial translocation is believed to be the primary mechanism driving chronic inflammation in HIV-1-infected individuals [11].

HIV-1 infection is characterized by continuous activation, rapid turnover, and activation-induced cell death of CD4^+^ and CD8^+^ T cell populations [11, 14]. The degree of immune activation represents an independent and more powerful predictor of disease progression than viral load [15].The destruction of supporting lymphoid tissue and activation-driven exhaustion of CD4^+^ T cell regenerative capability ultimately leads to the collapse of CD4^+^ T cell homeostasis [11, 14]. Despite significant effort, the precise mechanism underlying chronic T-cell activation in HIV-1 infection remains unknown. Accumulated evidence indicates that impairment of mucosal barrier function and resulting translocation of bacterial lipopolysaccharide (LPS) and other microbial antigens to the systemic compartment represents the primary mechanism driving continuous activation of CD4^+^ and CD8^+^ T cells in HIV-1 infection [16, 17]. Supporting this view is the fact that limited translocation of LPS is observed in chronically infected sooty mangabeys possibly explaining the low pathogenicity of SIV infection in its natural host [16]. Experimentally induced immune activation in natural hosts of SIV by administration of LPS results in significant increases in viral replication and CD4^+^ T cell depletion [18].

HIV-1-infection is associated with multiple aberrancies in humoral responses to previously as well as newly encountered antigens [19, 20]. It is well established that HIV-1 infection results in polyclonal B cell activation, hypergammaglobulinemia, reduction of memory B cell frequency, and an increase in the frequency of circulating immature B cells [21, 22]. Most of these changes occur concomitantly with the initial depletion of CD4^+^ T cells at mucosal tissues [20, 23]. However, relatively little is known regarding the effect of HIV-1 infection on the function of mucosal B cells. A near complete elimination of memory CD4^+^ T cell population in GALT during the acute stage of HIV-1 infection results in an absence of important regulatory and effector functions these cells play in the control of immune responses to environmental antigens and commensal bacteria on one side and infecting pathogens on the other [11, 24, 25, 26]. Since CD4^+^ T cells are required for immunoglobulin isotype switching and somatic hypermutation of B cells in mucosal lymphoid tissues, depletion of this vital population is likely to have a profound effect on the host’s ability to react to the changing antigenic variety at mucosal barriers [7, 11]. Altered production of mucosal antibodies may interfere with the essential role they play in the regulation of immune responses to common microbial and food antigens [25, 26]. Increased translocation of microbial products and whole bacteria is observed in SIV-infected macaques [12] and in HIV-1-infected patients with low levels of HIV-1-specific IgA antibodies [27]. It is likely that the perturbation of production of mucosal antigen-specific antibodies in HIV-1-infected individuals accelerates the translocation of bacteria, bacterial products, and other environmental antigens into the systemic compartment and significantly contributes to chronic inflammation in HIV-1 infection. Within this context, we addressed: a) whether the levels of antibodies specific to microbial and food antigens encountered at mucosal surfaces are altered in HIV-1-infected individuals with varying CD4^+^ T cell counts, b) whether the humoral responses to these antigens are differentially affected depending on the compartment (systemic versus mucosal), antigen type (bacterial, food, and yeast), and predominant isotype of the antibody, and c) whether a systemic expansion of antibodies to mucosal antigens significantly contributes to the hyperglobulinemia observed in HIV-1-infected individuals.

We present evidence of a significant increase of IgM levels and a decrease of IgG/IgM and IgA/IgM ratios of antibodies specific to mucosal microbial antigens in intestinal secretions of antiretroviral therapy (ART) -treated HIV-1-infected individuals indicative of a lack of help for class switch recombination. Altered isotype response to environmental antigens characterized by the shift from IgA to IgM may have significant consequences by promoting local inflammation and mucosal barrier damage [28]. Furthermore, this study demonstrates that HIV-1-infected individuals with low CD4^+^ T cell counts exhibit reduced levels of plasma IgG specific to antigens derived from Proteobacteria and Firmicutes. Reduced levels of microbiota-specific IgG and IgA may facilitate transepithelial translocation and systemic dissemination of mucosal microbial antigens and contribute to the chronic inflammation observed in HIV-1-infected individuals.

## Results

### HIV-1 infection is associated with a significant increase in total IgG and IgA levels in plasma and IgM levels in intestinal secretions

**Fig 1:**
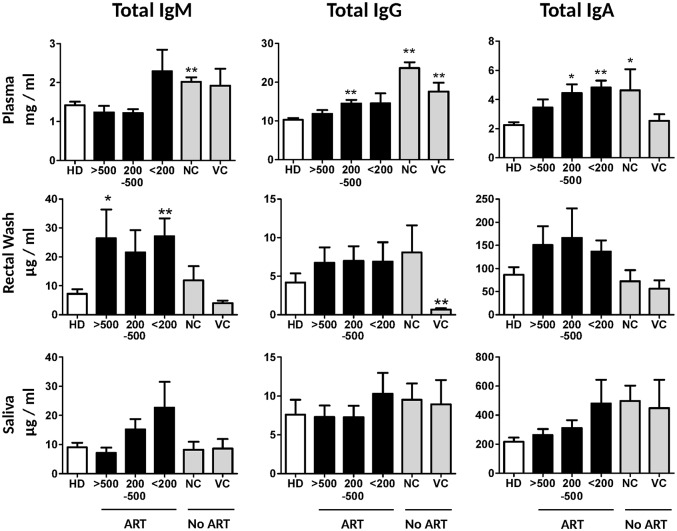
Total immunoglobulin levels in plasma and mucosal secretions of HIV-1-infected individuals and uninfected controls. Total levels of immunoglobulins were determined in plasma, rectal wash, and saliva of uninfected controls (n = 25), HIV-1-infected ART-treated patients with CD4^+^ T cell count > 500 (n = 24), 200–500 (n = 23), and < 200 (n = 16) per μl of blood, ART-untreated individuals not controlling viremia (non-controllers, NC; viral load 3,420–263,000 HIV-1 RNA copies per ml of blood, n = 9) and individuals spontaneously controlling viremia despite the absence of ART (viral controllers, VC; viral load < 1,150 of HIV-1 RNA copies per ml of blood, n = 9). Error bars represent SEM, statistical significance relative to seronegative donors was determined using Mann-Whitney *U* test (* *p* < 0.05; ** *p* < 0.01).

To analyze the levels of total and antigen-specific antibodies in systemic circulation and mucosal secretions in HIV-1 infection, plasma, rectal wash, and saliva samples were collected from 63 HIV-1-infected individuals on antiretroviral therapy (ART), 9 individuals not receiving ART who displayed open viremia (non-controllers; NC; viral load 3,420–263,000 HIV-1 RNA copies per ml of blood), 9 individuals spontaneously controlling viremia at low levels despite the absence of ART (viral controllers; VC; viral load < 1,150 HIV-1 RNA copies per ml of blood), and 25 HIV-1-seronegative controls (study cohort characteristics are listed in Table 1). As expected, the dominant immunoglobulin isotypes were IgG in plasma and IgA in rectal fluid and saliva. HIV-1 infection was associated with a significant increase of systemic IgG (Fig 1). In addition, HIV-1 patients with low CD4^+^ T cell counts displayed plasma IgA hypergammaglobulinemia. There was a negative correlation between CD4^+^ T cell count and total IgA in plasma of HIV-1-infected individuals (R = -0.4; *p* = 0.0002; Spearman rank order test). Non-controlling viremic individuals (NC) displayed an increase in all immunoglobulin isotypes. Plasma IgA and IgG levels inversely correlated with CD4^+^ T cell count (R = 0.41, *p* = 0.0002, and R = 0.29, *p* = 0.009, respectively). In intestinal secretions, ART-treated HIV-1-infected individuals displayed a profound increase of levels of total IgM (Fig 1).

**Table 1:** Study population.

	Individuals	Gender	Mean Age	Race	CD4^+^ cell	Viral load
	#	(M/F)	(rang)	W/AA^a^	(cells/μl)^c^	(copies/ml)^c^
HIV (-) Controls	25	13 / 12	36 (27–48)	11/14		
ART-treated						
CD4^+^ cells >500	24	12 / 12	43 (27–57)	8 / 14^b^	813 (517–1688)	228 (<50–412)
CD4^+^ cells 200–500	23	11 / 12	44 (22–59)	5 / 18	354 (207–498)	2,846 (<50–50,363)^d^
CD4^+^ cells <200	16	5 / 11	40 (28–57)	5 / 11	120 (22–183)	8,799 (<50–109,000)^e^
ART-naïve						
Non-controllers (NC)	9	3 / 6	39 (25–55)	2 / 7	475 (330–614)	69,827 (3,420–263,000)
Viral controllers (VC)	9	4 / 5	42 (25–53)	2 / 7	580 (271–895)	279(<50–1,150)

Notes:

^a^ W/AA: White/ African American;

^b^ One Asian; one not determined;

^c^ mean (range);

^d^ 2 of 23 individuals were not controlling viremia despite ART (14,200 and 50,363 HIV-1 RNA copies per ml of blood, respectively);

^e^ 2 of 16 individuals were not controlling viremia despite ART (17,800 and 109,000 HIV-1 RNA copies per ml of blood, respectively).

### Protein microarray of plasma humoral responses to antigens derived from intestinal microbiota indicates reduced systemic IgG responses to Proteobacteria and Firmicutes but not Bacteroidetes in HIV-1-infected individuals with low CD4^+^ T cell count

**Fig 2:**
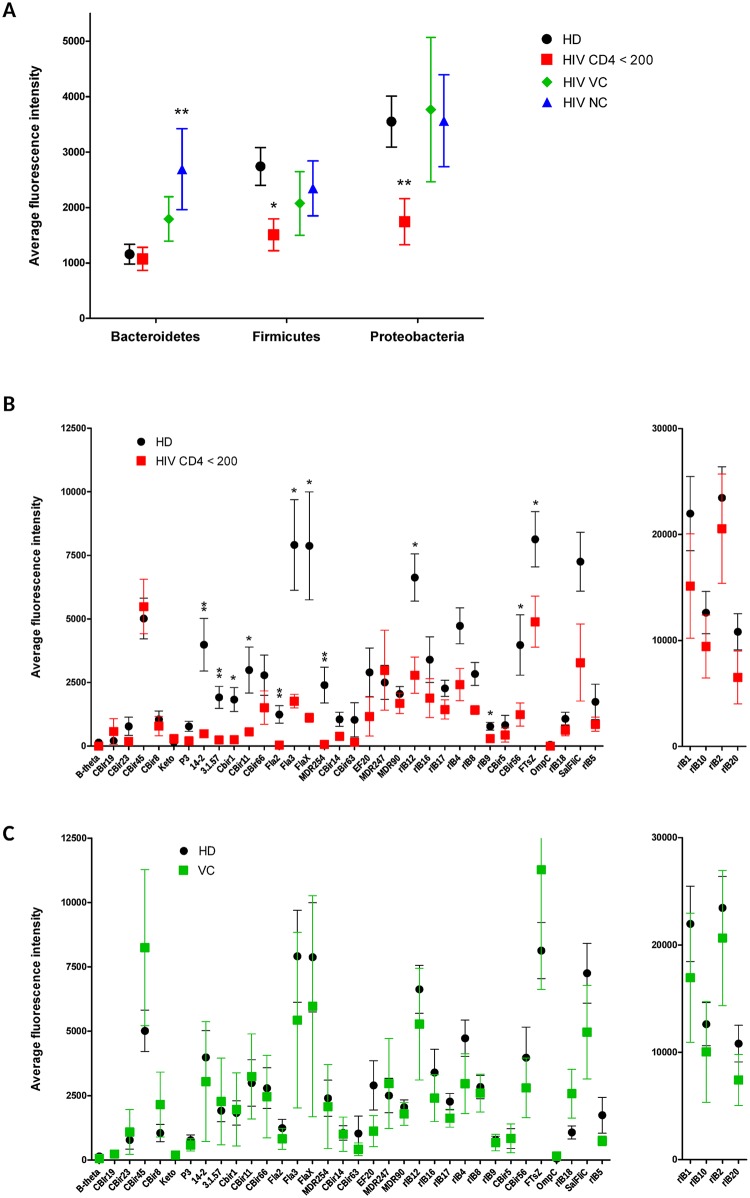
Protein microarray analysis of plasma IgG responses specific to intestinal microbial antigens. A) Average reactivity to antigens from different phyla. B, C) Comparison of responses to individual mucosal microbial antigens in healthy donors versus ART-treated HIV-1-infected individuals with less than 200 CD4^+^ T cells / μl of blood (HIV-1 CD4 < 200) (B) or ART-naïve individuals spontaneously controlling HIV-1(C). Data are presented as means ± SEM; * *p* < 0.05; ** *p* < 0.01; Mann-Whitney *U* test.

To investigate a wide range of humoral immune responses to intestinal microbiota in systemic circulation, we developed a novel protein microarray based on protein antigens derived from microbiota shown to be immunogenic in mice and humans [29]. The antigens represent a diverse set of proteins from the three most prominent phyla of the human gut microbiota: 8 Bacteroidetes antigens, 24 Firmicutes antigens, and 8 Proteobacteria antigens. 7 of these proteins are involved in metabolic functions, 13 are flagellin/ motility proteins, 6 associated with transcription/translation machinery, and 12 are cell surface proteins of various kinds. Consistent with previously described findings [29], while individual differences in the magnitude of response to individual antigens were observed, nearly all subjects exhibited a strong response to four universal antigens (rIB1, rIB10, rIB2 and rIB20) (Fig 2). Importantly, HIV-1-infected individuals with CD4^+^ T cell count < 200 / μl displayed significantly lower IgG responses to 11 of 38 mucosal antigens, mainly antigens derived from Proteobacteria and Firmicutes, but not those derived from Bacteroidetes (Fig 2A and 2B). Notably, significantly reduced responses to flagellins derived from Firmicutes were observed (flagellins 14–2, 3_1_57, CBir1, CBir11, CBir66, Fla 2, Fla 3, Fla X, and MDR254; for a detailed description of antigens please refer to [29]). In contrast, HIV-1-infected individuals spontaneously controlling infection (VC) displayed responses similar to uninfected controls (Fig 2C). ART-naïve viremic individuals (NC) displayed significantly increased response to Bacteroidetes-derived antigens (Fig 2A and S1 Fig).

### Systemic and mucosal levels of antibodies to representative bacterial, yeast, and food antigens

**Fig 3:**
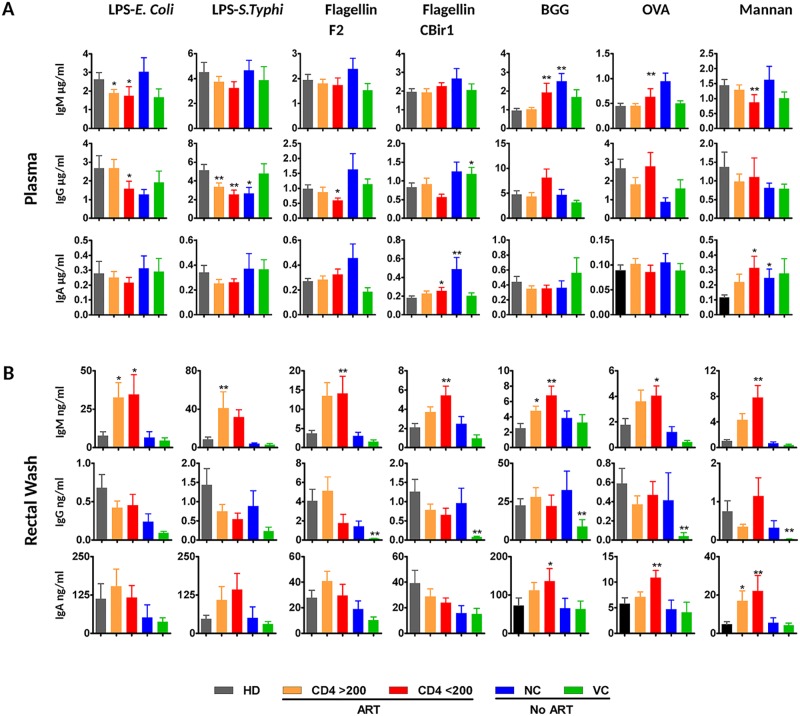
Concentrations of immunoglobulins specific for representative bacterial and food antigens in plasma and intestinal secretions of HIV-1-infected individuals. Individual plots represent the levels of immunoglobulins specific for lipopolysaccharide (LPS) produced by *Salmonella typhi* or *Escherichia coli*, flagellin F2 and CBir1, bovine gamma globulin (BGG), ovalbumin (OVA) and yeast mannan in plasma (A) and intestinal secretions (B). Error bars represent SEM, statistical significance relative to healthy donors was determined using Mann-Whitney *U* test (* *p* < 0.05; ** *p* < 0.01).

Protein microarray assay was optimized to address IgG levels in plasma; however, it does not provide information regarding the levels of other isotypes in plasma and mucosal secretions. Therefore, we next analyzed in detail the levels of all isotypes of antibodies specific to seven representative bacterial, yeast, and food antigens. The panel included lipopolysaccharide (LPS) antigens derived from *Escherichia coli* and *Salmonella typhi* since LPS represents a commonly used marker of microbial translocation and recently accumulated evidence indicates that it directly contributes to chronic inflammation in HIV-1 and SIV infections [16–18]. Furthermore, two flagellin proteins present in the protein microarray (flagellins CBir1 [*Roseburia faeces* strain M72] and F2 [*Roseburia intestinalis*]), two representative food antigens (bovine gamma globulin [BGG] and ovalbumin [OVA]), and one yeast antigen (mannan) were included. While *Escherichia coli* and *Roseburia* represent commensal organisms, *Salmonella typhi* is an example of a pathogenic organism. LPS was selected due to its prominent role in microbial translocation-induced immune activation [16]. Concentration per unit of plasma volume, i.e. concentration not normalized for the total level of Ig of the respective class, was analyzed by a modified ELISA assay. Antibodies to all selected antigens were detected in abundance in the blood of healthy individuals, confirming that humans readily recognize both microbial and food antigens and respond by robust humoral responses (Fig 3; heatmap representation is presented in S2 Fig). Importantly, the relative distribution of Ig isotypes was dependent on the type of antigen. While total plasma IgG prevailed over IgM by about ten fold, the ratio between specific IgG and IgM was about 5:1 for food antigens and 1:1 for bacterial and yeast antigens. Thus, the humoral response to food antigens was dominated by IgG while the response to intestinal microbial antigens was distributed equally between IgG and IgM isotypes. HIV-1-infected individuals with low CD4 T cell counts displayed decreased IgG response to most, but not all microbial antigens and increased IgM response to food antigens. In HIV-1-infected individuals with low CD4 T cell counts, significantly reduced plasma IgG level to flagellin F2 was detected confirming the results of the protein microarray assay; the decrease of IgG specific to CBir1 has not reached statistical significance.

In intestinal secretions, ART-treated HIV-1-infected individuals displayed a robust increase in levels of IgM specific to all types of mucosal antigens (Fig 3B). In addition, these individuals exhibited an increase in IgA responses to food antigens and yeast mannan. Viral controllers displayed a significant decrease in levels of IgG specific to most mucosal antigens compared to both ART-treated and untreated subjects. The data obtained in saliva samples displayed high variability diminishing the power of statistical analyses (S3 Fig). However, there was a negative correlation between CD4^+^ T cell count and total IgM in saliva (R = -0.34; *p* = 0.002) as well as IgM specific to flagellin CBir1 (R = -0.32; *p* = 0.004), flagellin F2 (R = -0.35; *p* = 0.002), OVA (R = -0.38; *p* = 0.0005), and BGG (R = -0.33; *p* = 0.003) in HIV-1-infected individuals. It should be noted that 2 of 23 individuals in CD4 200–500 group and 2 of 16 individuals in the CD4 < 200 group did not control viremia despite ART (Table 1). Total or antigen-specific antibody levels in the individuals not controlling viremia despite ART did not significantly differ from those of others in the respective groups. There was no significant association between the total or specific antibody levels and age or gender of the participants.

### Levels of microbial and food-specific antibodies normalized to total Ig level of the respective isotype

Expression of specific antibody levels in terms of absolute concentration is affected by high variabilities in the levels of total Ig in various secretions [30]. While the levels of Ig of the three major isotypes vary in sera within 0.5 log levels, in external secretions such as intestinal secretions the variability may reach 3 log difference [30]. It is therefore informative to express the levels of specific antibodies in normalized values relative to total Ig level. Importantly, if HIV-1 hypergammaglobulinemia was driven by non-specific B cell activation, it would be expected that normalized antigen-specific levels would follow total Ig levels and their relative concentration would, therefore, remain unaffected. However, the data presented in S4 Fig provide evidence invalidating this hypothesis. All three isotypes of plasma antibodies specific to most microbial and food antigens were present at lower relative concentrations in HIV-1-infected individuals compared to healthy subjects. Thus, HIV-1 infection is associated with a relative decrease rather than an increase of systemic humoral responses to antigens that are frequently encountered at mucosal surfaces. The notable exception to this rule is plasma IgM response to food antigens, in particular BGG. In intestinal secretions, there was no significant difference in specific IgM levels between HIV-1-infected and uninfected individuals. In general, plasma, gastrointestinal, and oral antibody responses followed significantly different patterns suggesting an independent humoral regulation in these compartments.

### HIV-1 infection is associated with changes in the relative ratios of Ig isotypes of total and mucosal antigen-specific antibodies

**Fig 4:**
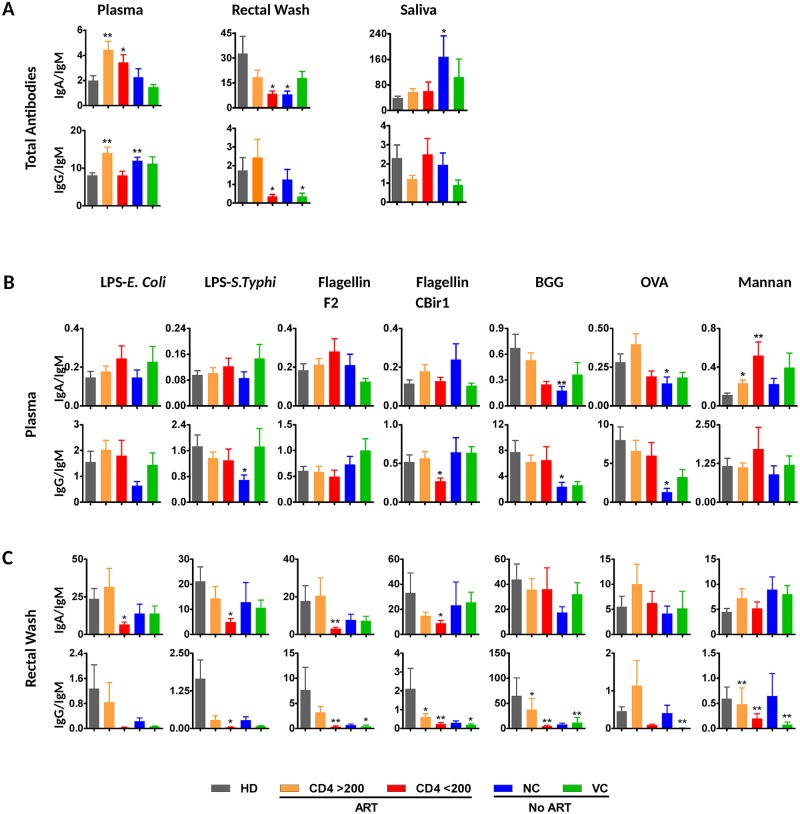
IgA/IgM and IgG/IgM ratios specific for antigens derived from intestinal microbiota are significantly decreased in intestinal secretions of HIV-1-infected individuals. IgA/IgM and IgG/IgM ratios of total (A) and antigen-specific immunoglobulin in plasma (B) and rectal washes (C). Error bars represent SEM, statistical significance relative to healthy donors was determined using Mann-Whitney *U* test (* *p* < 0.05; ** *p* < 0.01).

**Fig 5:**
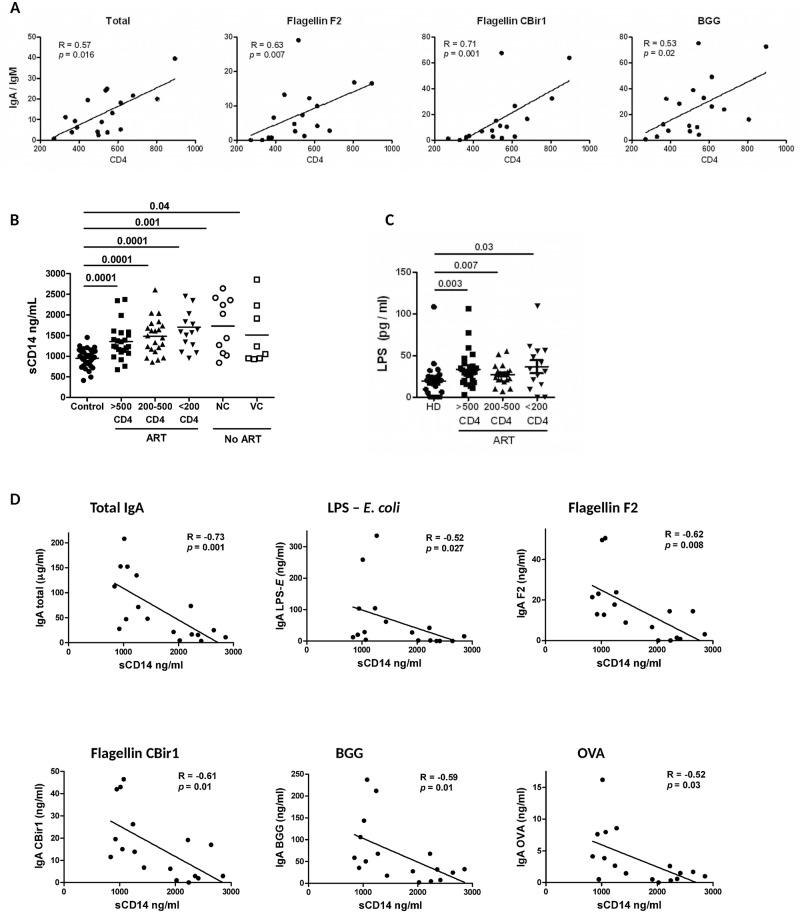
Correlation between the levels of plasma and intestinal IgG and IgA specific for microbial and food antigens, CD4^+^ T cell count, and plasma levels of sCD14. A) Total and mucosal antigen-specific IgA/IgM ratios in intestinal secretions of individuals not treated with ART (NC and VC) correlate with CD4^+^ T cell count / μl of blood. Correlations with LPS—*E*. *coli*, LPS—*S*. *typhi*, and yeast mannan antigens showed a similar trend but did not reach statistical significance. B, C) Plasma sCD14 and LPS levels are significantly increased in HIV-1-infected ART-treated and ART-naïve subjects. D) Rectal wash levels of total and mucosal-antigen specific IgA negatively correlate with plasma sCD14 in ART-naïve HIV-1-infected individuals. Correlations were performed using Spearman rank order test; solid lines represent linear regression analysis.

HIV-1 infection imparts opposite effects on Ig isotype ratios in plasma versus intestinal secretions. While an increase of IgA/IgM and IgG/IgM isotype ratios of total antibodies was observed in plasma of HIV-1 subjects compared to seronegative controls, a sharp decrease of these ratios was identified in a rectal wash of HIV-1-infected subjects with < 200 CD4^+^ T cells / μl (Fig 4A). Importantly, with respect to mucosal antigen-specific antibodies, a significant decrease of IgG/IgM ratios and IgA/IgM ratios was observed in a rectal wash of HIV-1-infected individuals (Fig 4C). Although the underlying causes of this decrease are unclear and may be multifactorial, the observation is consistent with a disruption of isotype switch mechanisms in GALT. To address whether the dysregulation of humoral responses is associated with CD4^+^ T cell depletion, mucosal antigen-specific IgA/IgM ratios were analyzed with respect to CD4^+^ T cell count. IgA/IgM ratios in intestinal secretions of all ART-naïve patients positively correlated with blood CD4^+^ T cell count for total (R = 0.57; *p* = 0.016), flagellin F2 (R = 0.63; *p* = 0.007), flagellin CBir1 (R = 0.71; *p* = 0.001), and BGG (R = 0.53; *p* = 0.02; Fig 5A; Spearman rank order test). In ART-treated individuals, the correlations between IgA/IgM ratios and CD4^+^ T cell counts did not reach statistical significance, although a similar trend was observed.

### Correlation between the levels of plasma and intestinal IgG and IgA specific for microbial and food antigens, plasma sCD14, and T and B cell activation

Consistent with previous reports, we observed a significant increase of markers of microbial translocation, sCD14, and LPS, in plasma of HIV-1-infected patients with high inter-individual variability (Fig 5B and 5C). In ART-treated patients, a negative correlation was observed between sCD14 and CD4^+^ T cell count (*p* = 0.02). Interestingly, in ART-naïve patients (combined NC and VC groups), significant negative correlations were observed between plasma sCD14 and total and mucosal antigen-specific levels of IgA in rectal fluid (Fig 5D). This observation suggests an association between lower mucosal production of specific IgA and an increase in microbial translocation. Although similar trends were observed in ART-treated patients, the correlations did not reach statistical significance for most antigens. There was a marginally positive correlation between IgA specific to BGG and OVA and sCD14 in plasma (R = 0.26, *p* = 0.05, and R = 0.37, *p* = 0.005). The reason for the differences between ART-treated and untreated groups and between specific antigens is unclear. Consistent with previous studies [14], we observed a significant increase in the activation of CD8^+^ and CD4^+^ T cells in HIV-1-infected individuals, in particular in individuals with < 200 CD4^+^ T cell / μl of blood and ART-naïve non-controlling (NC) individuals (S5 Fig). CD8^+^ T cell, but not CD4^+^ T cell activation positively correlated with plasma sCD14 levels (*p* < 0.03). A negative correlation was also observed between plasma sCD14 and frequency of circulating IgD^-^CD27^+^ memory B cells (R = -0.38, *p* = 0.005).

## Discussion

**Fig 6:**
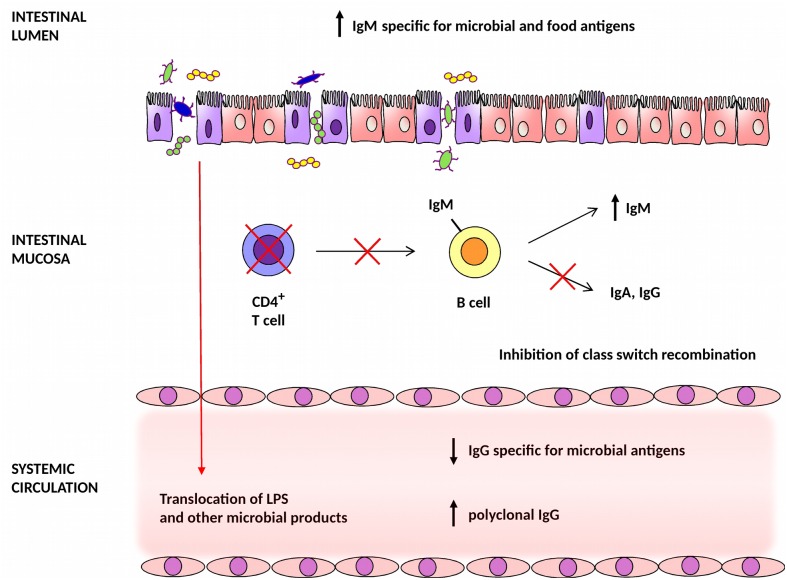
Dysregulation of systemic and mucosal humoral responses to microbial antigens in HIV-1-infected individuals. Depletion of CD4^+^ T cells in the intestinal mucosa of HIV-1-infected individuals reduces the capacity of mucosal B cells to undergo class switch recombination resulting in an increased production of IgM. Accumulated microbiota-specific IgM may exacerbate inflammatory processes by the formation of inflammatory immune complexes [28]. Epithelial cell apoptosis and decreased availability of antigen-specific IgG and IgA in mucosal secretions result in enhanced translocation of microbial products and whole bacteria across the intestinal mucosal barrier into the systemic circulation.

The study presented here reveals four major findings relevant to our understanding of systemic and mucosal humoral responses in HIV-1-infected individuals: (i) elevated levels of total and antigen-specific IgM in intestinal secretions; (ii) reduced IgG/IgM and IgA/IgM ratios of antibodies specific to microbial and food antigens in intestinal secretions; (iii) reduced IgG levels to antigens derived from Proteobacteria and Firmicutes in plasma; and (iv) a negative correlation between the levels of antigen-specific IgA in intestinal secretions and plasma levels of sCD14. Although the precise mechanisms underlying the observed reduction of IgG/IgM and IgA/IgM ratios of antibodies specific to mucosal microbial antigens and increase in intestinal IgM levels are presently unclear and are most likely multifactorial, the presented results are consistent with a lack of CD4^+^ T cell help for class switch recombination from IgM to other isotypes (Fig 6). In support of this observation, we and others have previously published that although the numbers of total IgA-producing cells in the intestines of HIV-1-infected individuals are unaltered, available evidence suggests a block in the induction and maintenance of de novo antigen-specific IgA response [12, 31, 32]. However, the precise nature of the underlying causes of the observed effects warrants further investigation in future studies addressing in detail the effect of HIV-1 infection of class-switching mechanisms in mucosal B cells.

The apparent defect in Ig isotype switching from IgM to IgG or IgA which results in the maintenance of vigorous IgM responses may have significant proinflammatory consequences. As demonstrated by Brandtzaeg and Tolo [28], when mucosally applied antigens interact with corresponding IgG or IgM antibodies with the formation of inflammatory immune complexes, activation of the complement cascade, the influx of polymorphonuclear leukocytes with ensuing mucosal barrier damage and enhanced absorption of by-stander antigens occurs. This is not the case of IgA antibodies which display a strong anti-phlogistic effect with no damage of mucosal tissues [31]. Accumulation of IgM-antigen complexes in intestinal mucosa may significantly contribute to the immunologically mediated damage of mucosal barrier function and enhanced microbial translocation.

A separate hypothesis tested in this study addressed whether the translocation of whole bacteria and bacterial products into the systemic circulation results in increased levels of antibodies specific to microbial antigens that may potentially drive hyperglobulinemia in HIV-1-infected individuals. Surprisingly, we have not observed an increase, but rather a decrease of systemic absolute and relative IgG levels specific to most antigens derived from intestinal microbiota. Thus, HIV-1-associated hypergammaglobulinemia is not driven by increased production of microbiota-specific antibodies. This observation sharply contrasts with increased microbiota-specific IgG levels observed in IBD patients [3–6]. The decrease in the levels of relative concentrations of antibodies of studied specificities is likely the result of a significant polyclonal expansion of immunoglobulin production of other, yet unknown specificities. The identity of antibodies responsible for systemic IgG and IgA hypergammaglobulinemia remains unclear. Interestingly, an increase of total and relative systemic humoral response to food antigens was observed, particularly in the IgM isotype. Likely the result of increased intestinal permeability, antibodies to food antigens may partially contribute to the hypergammaglobulinemia in HIV-1 infection.

Previous studies have demonstrated that HIV-1 disease progression is associated with a decrease of naturally occurring antibodies to the LPS core oligosaccharide, termed endotoxin-core antibodies (EndoCAb), that neutralize and clear LPS from circulation [16]. This contrasts with IBD patients who display an increase in EndoCAb levels [33]. Corroborating previous studies, we show that HIV-1-infected individuals with low CD4^+^ T cell counts display a decrease in both absolute and normalized levels of circulating IgG and a decrease in normalized levels of circulating IgA specific to LPS derived from *E*. *coli* and *S*. *typhi*. In addition, we show that the decrease is not specific to LPS, since the absolute levels of plasma IgG to flagellin F2 and normalized levels of IgG and IgA to flagellins F2 and CBir1 were lower in HIV-1-infected volunteers (Fig 3 and S4 Fig).

The mucosal lymphoid tissue is the site of induction, isotype switch recombination, and somatic hypermutation of B cells producing secretory IgA that consequently limits the penetration of microbial and food antigens through the intestinal epithelium [2, 24, 26, 34]. The regulation of isotype switch recombination and somatic hypermutation in B cells and transepithelial transport of IgA are dependent on stimulatory cytokines including IL-4, IFNγ, IL-5, IL-6, IL-10 and TGFβ produced by CD4^+^ T cells [35, 36]. Recent evidence suggests that germinal center B cell activation and selection is dependent on the interaction between CD4^+^CXCR5^+^PD-1^+^ follicular helper (T_FH_) cells and CD4^+^CXCR5^+^PD-1^+^FoxP3^+^Blimp-1^+^ follicular regulatory (T_FR_) cells [37–39]. T_FH_ cells have been identified as a major compartment for HIV-1 infection, replication, and production that is partially resistant to the effect of antiretrovirals [40]. Continuous infection and impairment of the function of T_FH_ cells may significantly contribute to dysregulation of mucosal humoral responses [41]. T_FR_ cells specialize in controlling the germinal center reaction through inhibiting the activation of non-specific B cells. Although not specifically addressed in this study, partial depletion or altered function of CD4^+^ T_FH_ and T_FR_ cells may be responsible for the observed dysregulation of mucosal humoral responses in HIV-1-infected individuals; this aspect warrants further investigation.

The data presented here confirm a low degree of interdependence of IgA responses to mucosal intestinal antigens between the intestinal and systemic compartments. This adds to the accumulated evidence that IgA in plasma and external secretions display a remarkable degree of mutual independence. IgA in plasma originates overwhelmingly from the bone marrow and to a lesser degree from lymph nodes and spleen; in contrast, secretory IgA is produced locally in mucosal tissues. VH repertoire of IgA-producing plasma cells in mucosal vs. systemic compartments is vastly different [42, 43]. Another interesting observation concerns a decrease of total as well as specific IgG antibodies in the rectal wash of individuals spontaneously controlling HIV-1 infection (Figs 1 and 3). Although the underlying reason is unclear, one possibility is that since IgG is produced systemically and enters mucosal secretions primarily via exudation from microvasculature, viral controllers display increased endothelial tightness in GALT microvasculature. The same mechanism would also limit the spread of microbial products via hepatic portal vein into the liver and systemic circulation, resulting in lower immune activation and viral proliferation. It is unclear whether this property would be inherent to spontaneously controlling subjects prior to infection or would arise as a result of a specific response to HIV-1 infection.

We observed reduced systemic IgG response to antigens derived from Proteobacteria and Firmicutes in individuals with low CD4^+^ T cell count and increased response to Bacteroidetes in viremic individuals. Previous studies have demonstrated decreased bacterial richness and phylogenetic diversity in HIV-1-infected individuals with CD4^+^ T cell count < 200 compared to subjects with CD4^+^ count >200 [44, 45]. Interestingly, multivariate analysis showed that *Ruminococcacaea*, *Clostridiaceae*, and *Enterobacteriaceae* families (first two belonging to Firmicutes and third to Proteobacteria phyla) are independently associated with the low CD4^+^ T cell count. *Enterobacteriaceae* consisted primarily of genus *Shigella* and closely related *Escherichia* species [44]. Similar results were obtained in SIV-infected rhesus macaques with low CD4^+^ T cell count [46]. Interestingly, Klase et al. recently demonstrated that Proteobacteria preferentially translocate from gut lumen to mesenteric lymph nodes and liver and the extent of translocation positively correlates with CD4^+^ T cell activation in the same tissue [47]. It is interesting to speculate that decreased response to specific members of Proteobacteria, and possibly Firmicutes, phyla combined with their altered relative abundance in gut lumen significantly contribute to chronic inflammation and disease progression in HIV-1 infection. Alternatively, since inflammation in the intestinal mucosa has been shown to support the growth of specific *Enterobacteriaceae* family members, it is also possible that HIV-1-induced inflammation promotes changes in the intestinal microbial ecosystem [48]. Interestingly, while only modest changes in the intestinal microbiome were observed as a result of experimental SIV infection of Asian macaques, substantial dysbiosis occurred after administration of antiretrovirals [47]. Although the mechanisms underlying the observed effect of ART on gut microbiome are unclear and warrant further investigation, this could potentially explain why the observed dysregulation of intestinal IgM production described here was more significant in ART-treated individuals than untreated HIV-1-infected controls.

Limitations of this study include cross-sectional design, a limited number of subjects in experimental groups (in particular in groups of individuals not treated with ART), and absence of analysis of cells isolated from mucosal tissues. Although the presented data indicate reduced competence of mucosal B cells for class switch recombination from IgM to other isotypes, the effect of HIV-1 infection on specific class-switching mechanism was not directly addressed. However, the presented findings are consistent with earlier studies describing B cell disturbances in GALT of HIV-1-infected individuals and SIV-infected macaques including intestinal B cell hyperactivity, destruction of gastrointestinal germinal centers in acute phase of infection, and specific blocking of intrafollicular but not extrafollicular Ig switch enzyme activation-induced deaminase [10, 32, 41, 49, 50].

In summary, decreased capacity of mucosal B cells to respond to newly occurring microbial variants due to the block of class switch recombination described here may facilitate trans-epithelial translocation and systemic dissemination of microbial antigens in HIV-1-infected individuals. Since elevated microbial translocation is recognized as a critical mechanism driving chronic inflammation in HIV-1-infected individuals despite successful control of viral replication by ART, disruption of normal humoral response to changing antigenic variety at mucosal surfaces may have a profound effect on disease progression.

## Methods

### Ethics statement

All procedures were performed in accordance with guidelines set forth by the University of Alabama at Birmingham (UAB) Institutional Review Board (IRB) and the study was approved by UAB IRB committee. All volunteers provided written informed consent.

### Study population

A total of 106 human subjects were recruited at the UAB 1917 AIDS clinic. 25 age- and gender-matched HIV-1-seronegative volunteers were recruited as controls (Table 1). HIV-1-infected patients treated with ART were divided into three groups based on their CD4^+^ T cell count per μl of blood, > 500 (n = 24), 200–500 (n = 23), and < 200 (n = 16). 2 of 23 individuals in CD4 200–500 group and 2 of 16 individuals in the CD4 < 200 group did not control viremia despite ART (Table 1). Patients not treated with ART were divided into a group of individuals not controlling viremia (non-controllers; NC; viral load 3,420–263,000 HIV-1 RNA copies per ml of blood; n = 9) and individuals spontaneously controlling viremia at low level despite the absence of ART (viral controllers; VC; viral load <1,150 of HIV-1 RNA copies per ml of blood; n = 9). 4 of 9 viral controllers were classified as elite controllers (viral load <50 HIV-1 RNA copies per ml of blood in the absence of ART).

### Sample collection procedures

Plasma, serum, rectal wash, and saliva were collected from each study subject at the Alabama Vaccine Research Clinic at UAB with the standard operating procedures for collection of blood and mucosal specimens [30]. Peripheral blood was collected with ACD solution A venous blood collection tubes (BD, Franklin Lakes, NJ). Rectal wash was collected by instillation of 50 ml saline into the rectum, and after 5 minutes retaining the fluid was expelled into the collection device containing protease inhibitors aprotinin, leupeptin, antipain and pepstain (Sigma), sodium azide and PMSF (Sigma, St. Louis, MO) as described [51, 52]. The expelled fluid was filtered and centrifuged at 2000g for 10 minutes. Unstimulated parotid saliva was collected with an intraoral plastic cup (Schaeffer cup) fitted in the participant’s buccal vestibule over the opening of the Stensen’s duct of the parotid gland [30]. The supernatants from centrifuged samples were aliquoted and cryopreserved at -70°C until used.

### ELISA for total and antigen-specific immunoglobulins

Bovine gamma globulin (BGG), ovalbumin (OVA, from chicken egg white, Grade VII), mannan (*S*. *cerevisise*), lipopolysaccharides (LPS; from *S*. *typhi* and *E*. *coli*) were obtained from Sigma. A4 Fla2 and CBir1 flagellin were provided by Dr. Charles O. Elson [4, 53]. Briefly, antigens were dissolved in PBS were coated in 96 well MaxiSorp immuno plates (Nalge Nunc international, Rochester, NY), at previously determined optimal concentrations. For total immunoglobulin measurement and standard curves of indirect assessment of antigen-specific antibodies, plates were coated with polyclonal goat F(ab’)_2_ fragments of anti-human IgA, IgG, or IgM (Jackson ImmunoResearch, West Grove, PA), 1 μg/ml in PBS, overnight at 4°C. Plates were washed with PBS containing 0.05% Tween-20 (PBST) and blocked with 5% goat serum (Caisson Laboratories, North Logan UT) in PBST for IgA and IgM, or 1% BSA (Millipore, Kankakee, IL) for IgG, for 2 hours at room temperature. Samples of plasma or external secretions in PBST with 2% goat serum for IgA and IgM or with 1% BSA for IgG were incubated overnight at 4°C. After washing, biotinylated F(ab’)_2_ fragments of goat anti-human IgA, IgM and IgG (Biosource International, Camarillo, CA) were added for 1 h. Levels of bound antibodies were detected using streptavidin-conjugated peroxidase (ZYMED Laboratories, Carlsbad, CA) and SureBlue TMB peroxidase substrate (KPL, Gaithersburg, MD). Optical density values were determined using E-312e plate reader (Bio-Tek Instruments, Winooski, VT) and the concentration was calculated using Delta Soft 3 software. Concentrations of total and specific antibodies of all isotypes in plasma, rectal wash and saliva were standardized against standard human serum (Microgenics Corporation, Fremont, CA).

### Microbiota antigen microarray

The proteins were printed onto FAST 16 nitrocellulose pad slides (Whatman) using a MicroGrid II robot (Genomic Solutions) in duplicate in two different parts of the pad as described [29]. Each antigen was present in quadruplicate. Slides probed with human sera at 1:100 dilution, washed, and incubated with Alexa 647- or Alexa 546-labeled goat anti-human IgG or IgA (KPL). The slides were read in an Axon GenePix 4000B dual laser microarray reader. GenePix Pro 6.0 software was used to determine the net median pixel intensities for each individual feature (antigen spots) from a set of 10 measurements/feature. A median net digital fluorescence unit (DFU) for each feature represents the median values from 4 replicate antigen features on each array. Statistical analysis of data was performed with R statistical package or GraphPad Prism using appropriate tests to compare values between groups. Individual antigens included in the microarray were as previously described [29].

### Human sCD14 and LPS determination

Human sCD14 in plasma was determined using human sCD14 Immunoassay kit (R&D Systems, Minneapolis, MN) according to manufacturer’s protocol. Levels of LPS were determined using the Limulus Amebocyte Lysate (LAL) assay (QCL-1000; Lonza, Walkersville, MD).

### Flow cytometric analysis of T and B cell subsets

0.5 x 10^6^ isolated PBMCs were stained with anti-CD3-PacBlue, CD4-APC-Alexa Fluor750, CD8-PerCP-CY5.5, CD27-APC, CD45RO-PE, CD57-FITC, and CD38-PE-Cy7 (eBioscience) to distinguish T cell subsets and levels of activation. B cell subsets and activation status was analyzed by staining with CD70-FITC, α-IgD-PE (BD), CD19-PE-Cy7, and CD27-APC (BD) antibodies. Appropriate isotype controls were used to determine the percentages of cells expressing the respective markers (Biolegend, San Diego, CA). Samples were analyzed on LSR-II flow cytometer (BD) and data analysis was performed using the FlowJo (FlowJo, LLC) and FACSDiva software (BD) as described [52, 54, 55].

### Statistical analysis

Correlations were performed using Spearman rank order test or by Pearson product-moment correlation test for populations that passed D'Agostino & Pearson omnibus normality test. Comparisons between experimental groups were performed using Mann-Whitney *U* test. GraphPad Prism 5 (GraphPad Software Inc., LaJolla, CA) statistical and graphing software package was used for data analysis. Bonferroni correction for multiple comparisons was applied as appropriate. A standard level of statistical significance α = 0.05 was used; all reported *p*-values are two-sided.

## Supporting Information

S1 Fig Protein microarray analysis of plasma IgG responses specific to intestinal microbial antigens. Comparison of responses to individual mucosal microbial antigens in healthy donors versus ART-naïve HIV-1-infected non-controlling (NC) individuals. Data are presented as means ± SEM; * p < 0.05; ** p < 0.01.
(TIF)

S2 Fig Heatmap representation of absolute concentrations of immunoglobulins specific for common mucosal bacterial and food antigens in plasma, rectal wash, and saliva of HIV-1-infected ART-treated and untreated individuals normalized to healthy donors. (TIF)

S3 Fig Concentrations of immunoglobulins specific for representative bacterial and food antigens in plasma and intestinal secretions of HIV-1-infected ART-treated and -untreated subjects. Individual plots represent the levels of immunoglobulins specific for lipopolysaccharide (LPS) expressed by *Salmonella typhi* or *Escherichia coli*, flagellins A4 FLA2 and CBir1, bovine gamma globulin (BGG), ovalbumin (OVA) and yeast mannan in saliva. Error bars represent SEM, statistical significance relative to seronegative donors was determined using Mann-Whitney *U* test (* *p* < 0.05; ** *p* < 0.01).
(TIF)

S4 Fig Normalized levels of immunoglobulins specific for common mucosal bacterial and food antigens in plasma, rectal wash, and saliva of HIV-1-infected ART-treated and untreated individuals. Levels of specific immunoglobulins in plasma (A), rectal wash (B), and saliva (C) are presented as ng / μg of total immunoglobulin of the relevant class. Error bars represent SEM, statistical significance relative to healthy donors was determined using Mann-Whitney *U* test (* *p* < 0.05; ** *p* < 0.01).
(TIF)

S5 Fig CD38 expression on naïve (Tn), central memory (Tcm), effector (Tef), and effector memory (Tem) CD8^+^ (A) and CD4^+^ (B) T cells. (TIF)
