# Supplementary figures and images for: Dysregulation of Systemic and Mucosal Humoral Responses to Microbial and Food Antigens as a Factor Contributing to Microbial Translocation and Chronic Inflammation in HIV-1 Infection
Source: PLoS Pathog. 2017 Jan 26;13(1):e1006087. doi: 10.1371/journal.ppat.1006087 (PMC5268400; doi:10.1371/journal.ppat.1006087)

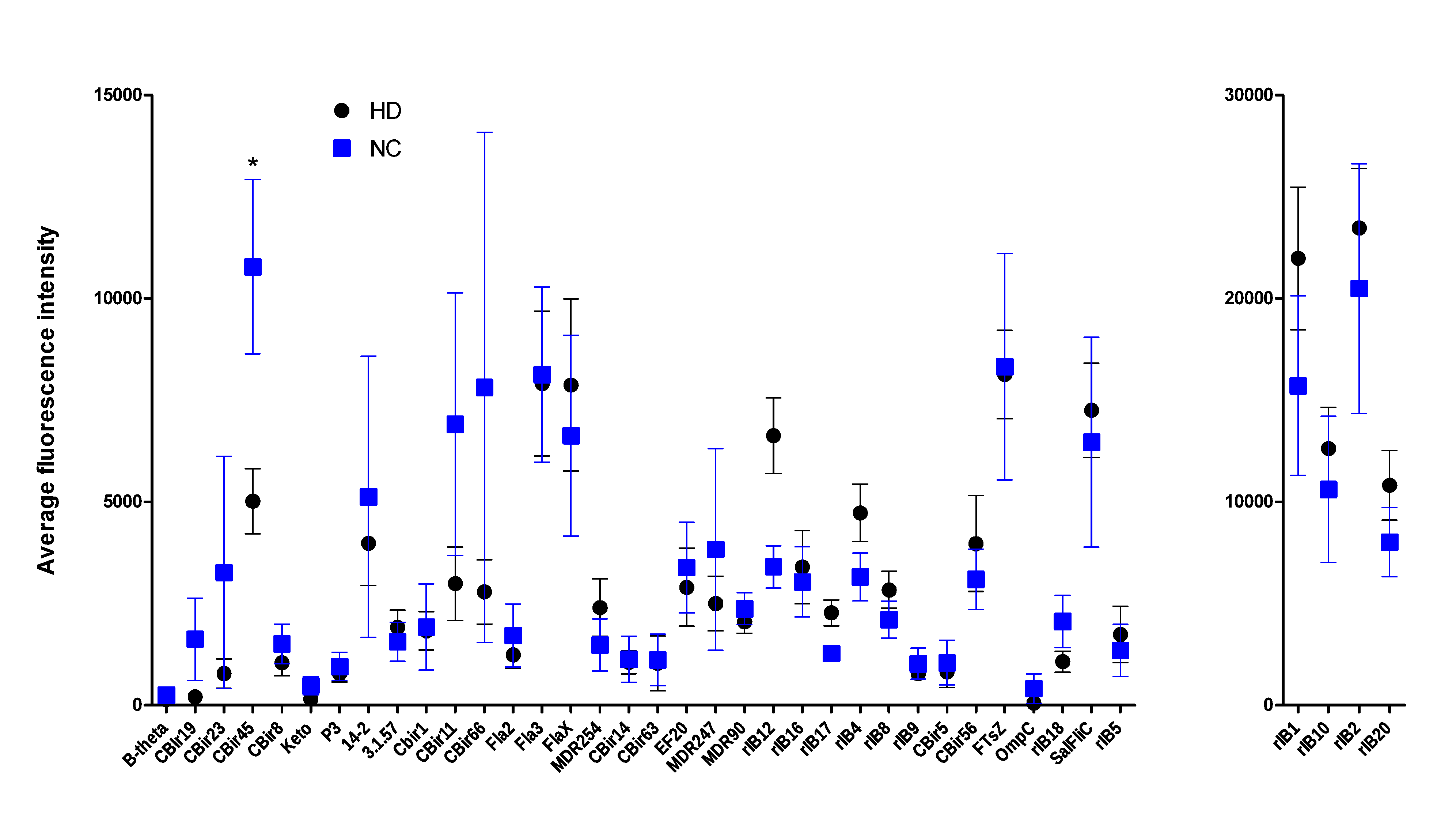

Supplement: S1 Fig — Comparison of responses to individual mucosal microbial antigens in healthy donors versus ART-naïve HIV-1-infected non-controlling (NC) individuals. Data are presented as means ± SEM; * p < 0.05; ** p < 0.01. (TIF) [file ppat.1006087.s001.tif]

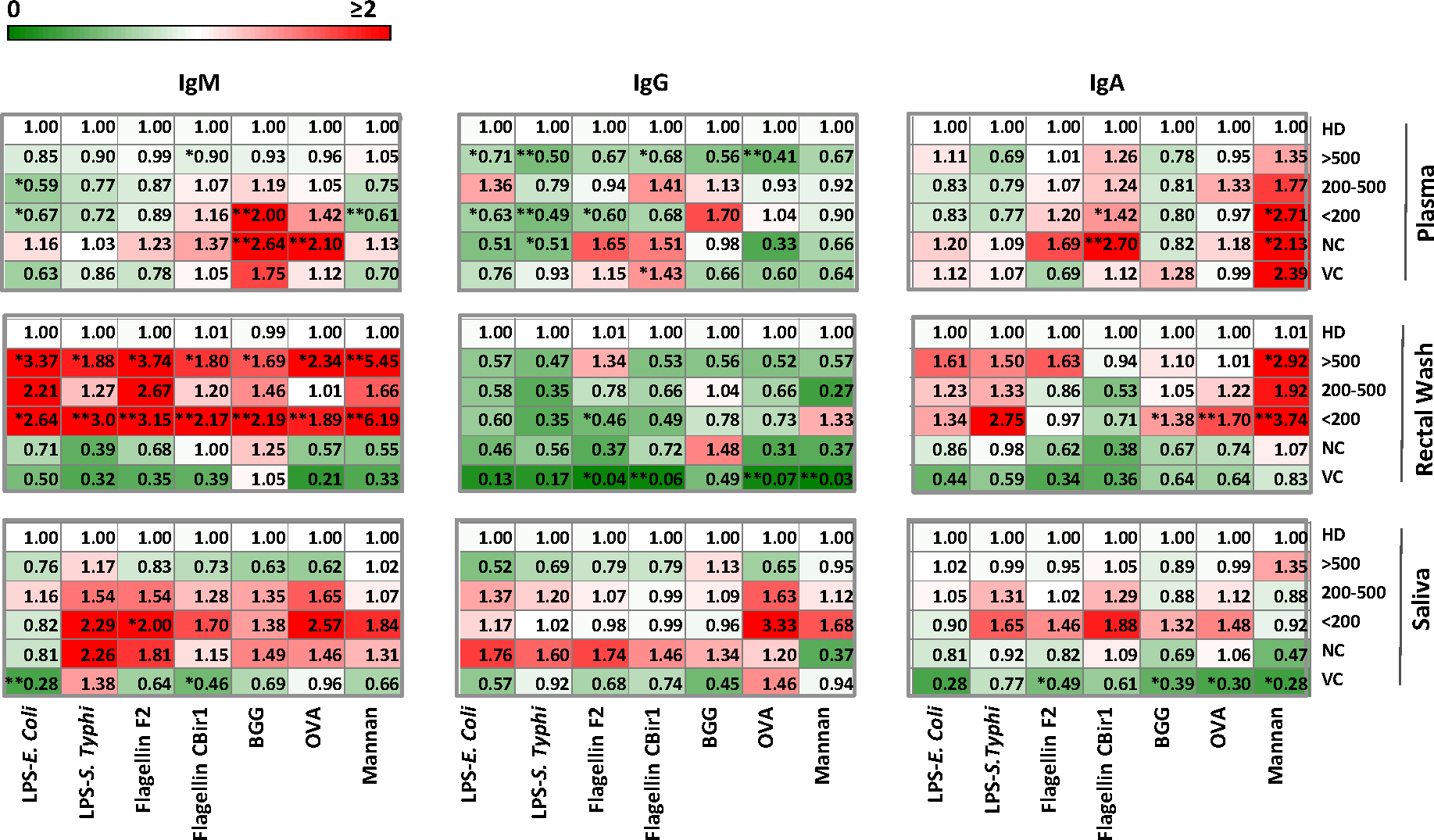

Supplement: S2 Fig — (TIF) [file ppat.1006087.s002.tif]

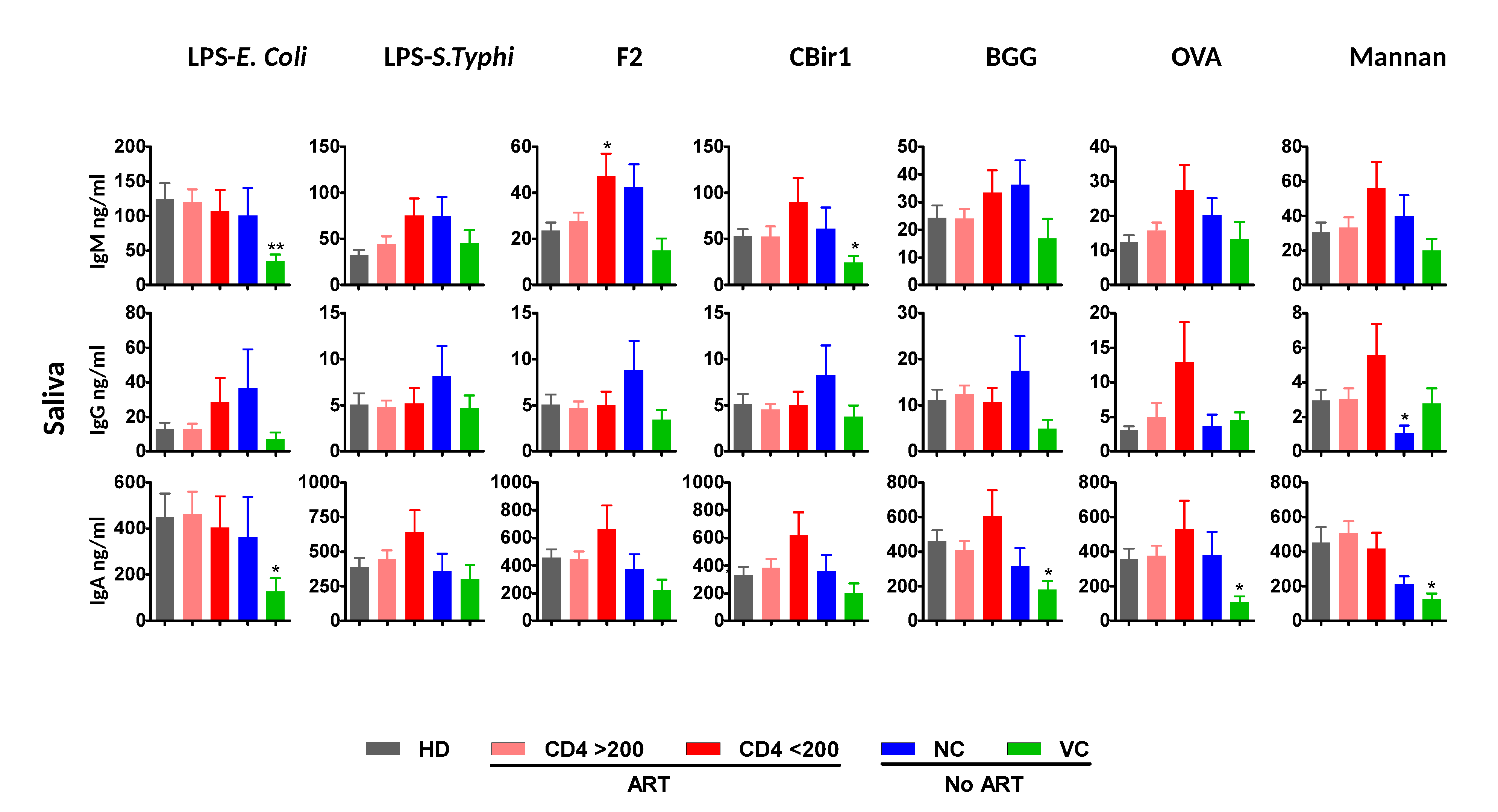

Supplement: S3 Fig — Individual plots represent the levels of immunoglobulins specific for lipopolysaccharide (LPS) expressed by Salmonella typhi or Escherichia coli, flagellins A4 FLA2 and CBir1, bovine gamma globulin (BGG), ovalbumin (OVA) and yeast mannan in saliva. Error bars represent SEM, statistical significance relative to seronegative donors was determined using Mann-Whitney U test (* p < 0.05; ** p < 0.01). (TIF) [file ppat.1006087.s003.tif]

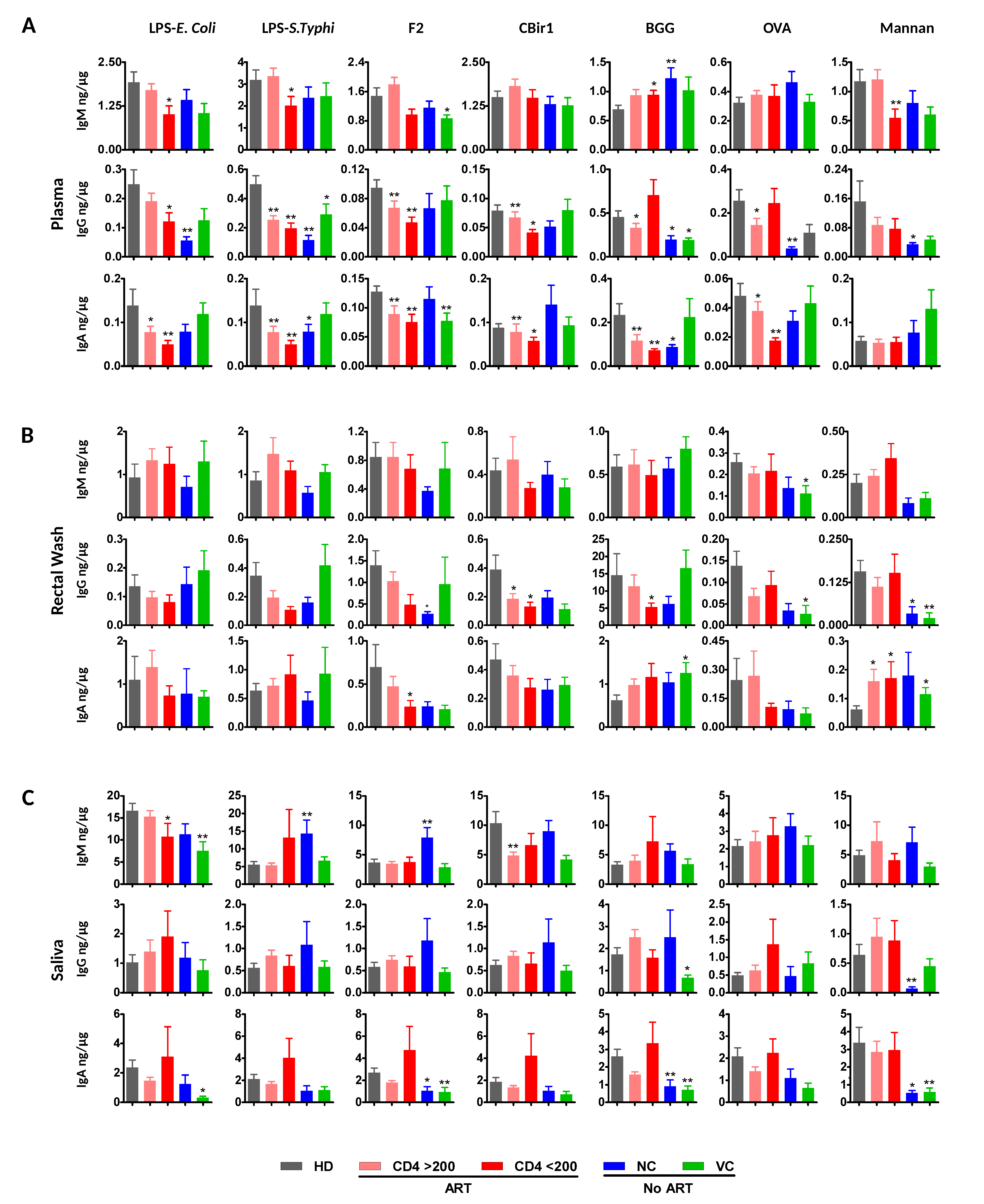

Supplement: S4 Fig — Levels of specific immunoglobulins in plasma (A), rectal wash (B), and saliva (C) are presented as ng / μg of total immunoglobulin of the relevant class. Error bars represent SEM, statistical significance relative to healthy donors was determined using Mann-Whitney U test (* p < 0.05; ** p < 0.01). (TIF) [file ppat.1006087.s004.tif]

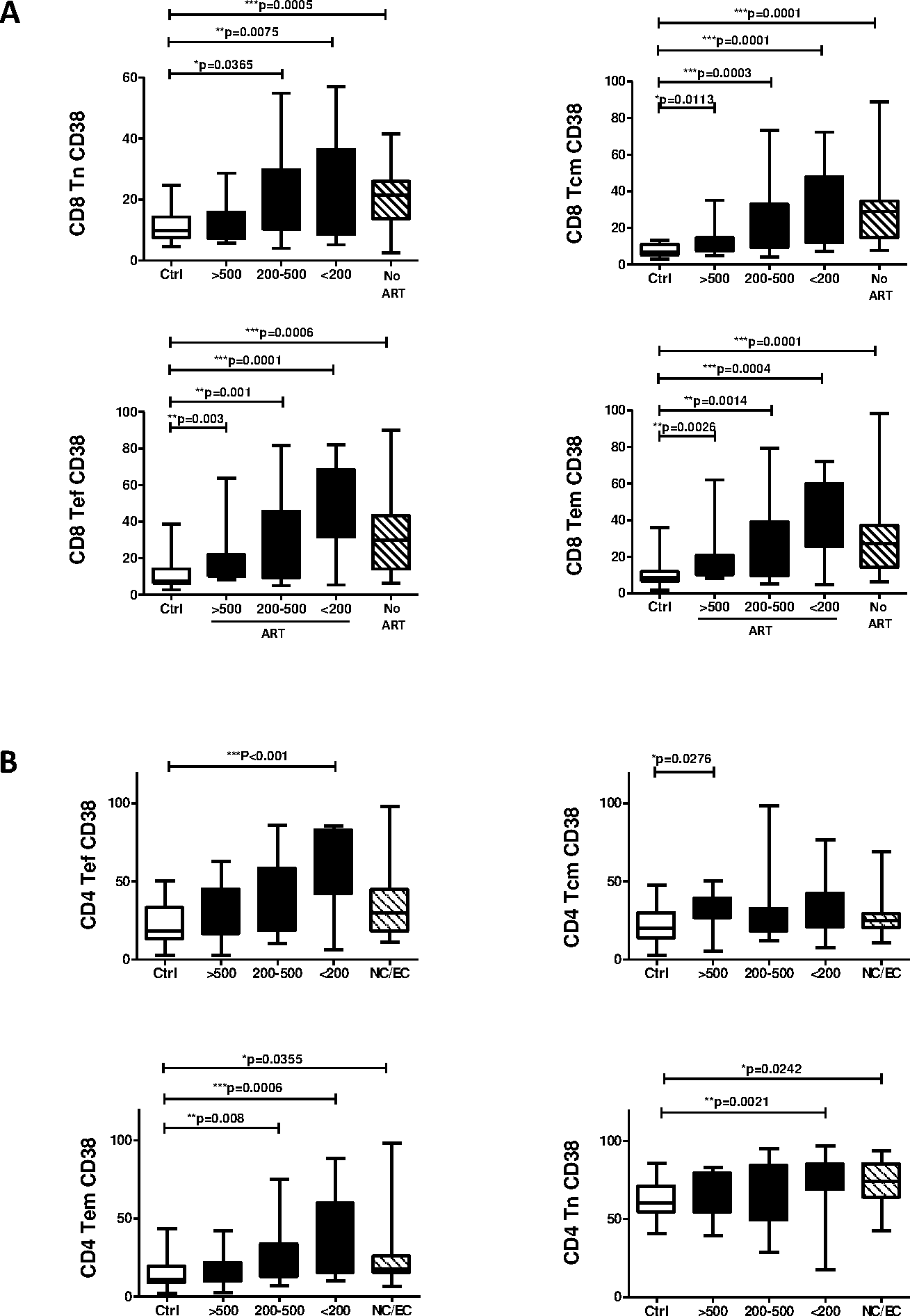

Supplement: S5 Fig — (TIF) [file ppat.1006087.s005.tif]
